# A towards-multidimensional screening approach to predict candidate genes of rheumatoid arthritis based on SNP, structural and functional annotations

**DOI:** 10.1186/1755-8794-3-38

**Published:** 2010-08-20

**Authors:** Liangcai Zhang, Wan Li, Leilei Song, Lina Chen

**Affiliations:** 1 Department of Biophysics, College of Bioinformatics Science and Technology; Harbin Medical University; Harbin, Hei Longjiang Province, China

## Abstract

**Background:**

According to the Genetic Analysis Workshops (GAW), hundreds of thousands of SNPs have been tested for association with rheumatoid arthritis. Traditional genome-wide association studies (GWAS) have been developed to identify susceptibility genes using a "most significant SNPs/genes" model. However, many minor- or modest-risk genes are likely to be missed after adjustment of multiple testing. This screening process uses a strict selection of statistical thresholds that aim to identify susceptibility genes based only on statistical model, without considering multi-dimensional biological similarities in sequence arrangement, crystal structure, or functional categories/biological pathways between candidate and known disease genes.

**Methods:**

Multidimensional screening approaches combined with traditional statistical genetics methods can consider multiple biological backgrounds of genetic mutation, structural, and functional annotations. Here we introduce a newly developed multidimensional screening approach for rheumatoid arthritis candidate genes that considers all SNPs with nominal evidence of Bayesian association (*BFLn > 0*), and structural and functional similarities of corresponding genes or proteins.

**Results:**

Our multidimensional screening approach extracted all risk genes (*BFLn > 0*) by odd ratios of hypothesis H_1 _to H_0_, and determined whether a particular group of genes shared underlying biological similarities with known disease genes. Using this method, we found 6614 risk SNPs in our Bayesian screen result set. Finally, we identified 146 likely causal genes for rheumatoid arthritis, including CD4, FGFR1, and KDR, which have been reported as high risk factors by recent studies. We must denote that 790 (96.1%) of genes identified by GWAS could not easily be classified into related functional categories or biological processes associated with the disease, while our candidate genes shared underlying biological similarities (*e.g*. were in the same pathway or GO term) and contributed to disease etiology, but where common variations in each of these genes make modest contributions to disease risk. We also found 6141 risk SNPs that were too minor to be detected by conventional approaches, and associations between 58 candidate genes and rheumatoid arthritis were verified by literature retrieved from the NCBI PubMed module.

**Conclusions:**

Our proposed approach to the analysis of GAW16 data for rheumatoid arthritis was based on an underlying biological similarities-based method applied to candidate and known disease genes. Application of our method could identify likely causal candidate disease genes of rheumatoid arthritis, and could yield biological insights that not detected when focusing only on genes that give the strongest evidence by multiple testing. We hope that our proposed method complements the "most significant SNPs/genes" model, and provides additional insights into the pathogenesis of rheumatoid arthritis and other diseases, when searching datasets for hundreds of genetic variances.

## Background

Rheumatoid arthritis is an inflammatory disease, primarily of the joints, with autoimmune features and a complex genetic component [1]. It arises from the underlying functional involvement of one or more mutated genes [1,2]. The essential challenge of rheumatoid arthritis is finding an effective screening approach to find candidate risk genes by their structural and functional similarity to known disease genes, and using them to develop new techniques for testing, diagnosis, and treatment [3-5].

When case-control datasets of complex diseases are available, genome-wide association studies (GWAS) have great power to detect genetic variants, especially if many markers are tested across the genome [6-8]. All published GWAS have led to the discovery of novel genes for complex diseases that differ between case and control groups. However, because of the arbitrary multiple testing used in these studies, genetic variants that confer a small disease risk but are of potential biological importance are likely to be missed using a "most significant SNPs/genes" approach [9,10]. To avoid the strict adjustment required in multiple testing, we developed a genome-wide Bayesian association method to test for association of a single SNP with a case-control phenotype. The Bayesian approach compares the probability of an association to the probability given no association. For complex diseases, discovering new bioinformatics strategies based on genome-wide Bayesian association methods that avoid the limitations of other study is vital.

Traditional statistical genetics aims to identify susceptibility genes based only on a statistical model without considering biological similarities between disease genes and likely causal genes. Proteins are essential parts of organisms and participate in virtually every cellular process. Most proteins fold into unique sequence arrangements and structures, and contribute to specific characteristics in diverse function sets. Proteins and genes that are responsible for complex diseases are often associated through similar sequences and structures [11-13], so candidate genes could be screened according to sequence, arrangement, and crystal structures that are similar to known disease genes. A support vector machine (SVM) is a machine learning algorithm based on Statistical Learning Theory that is commonly applied to resolve this problem [14-18]. Good classification effects can be obtained with only a few learning samples. Many studies [19-22] have demonstrated that disease genes with a specific phenotype share similar functionalities, and therefore, similarity in the functional annotations of these genes could be used to screen for candidate genes for a specific disease. A limited number of studies have used GWAS [23-25], function clustering algorithms [26-29], or machine learning methods based on structural genomics knowledge bases [30] to identify candidate genes for rheumatoid arthritis. When a set of candidate risk genes are acquired from case-control datasets of genetic variances, joint consideration of the structural and functional associations between candidate genes and a disease might provide additional insights into the results of traditional statistic genetics analysis for identifying candidate genes.

In this article, we hypothesized that underlying candidate genes harboring markers with minor or modest evidence of association could be identified through attributions they share with known disease genes, using multidimensional biological annotations such as gene sequence arrangement, crystal structure of encoded proteins, and similar biological pathways or mechanisms. Here, we introduce a newly developed multidimensional screening approach to predict candidate genes of rheumatoid arthritis based on SNPs, and structural and functional annotations. The rationale for performing our multidimensional candidate gene screen was the assumption that several genes, each modestly associated with a disease, may share sequence or structural pattern, and jointly participate in the same biological function to confer susceptibility. We used a genome-wide Bayesian association method to test for association between a case-control phenotype and a single SNP. To avoid the strict adjustment required for multiple testing, Bayesian approaches compare the probability of an association to the probability of no association. An SVM classifier was used to distinguish likely causal genes from non-disease genes by the sequence and crystal structural features of their proteins. Candidate genes were assumed to be disease genes if they were in the same functional categories or biological pathways associated with the pathogenesis. We carried out literature searches to verify our results, and compared them with traditional GWAS results to demonstrate the potential utility of this method.

## Methods

### Genetic Association Data of Rheumatoid Arthritis

Genotype frequencies of tested SNPs for case-control samples were downloaded from GAW16 online using the 500 K Affymetrix chip, from 868 cases and 1194 controls from the rheumatoid arthritis collection and normal samples http://www.gaworkshop.org/. Genotype frequencies were preprocessed to allele frequencies for each SNP.

### Gene Location and Disease Loci Data

Location information for human genes was from the NCBI genome database (downloaded on Mar 25, 2009). Disease loci information was gathered from the OMIM online database (downloaded on Mar 25, 2009) [31].

### Sequence and Crystal Structure Data

Linear-sequence items for all human genes were from the NCBI genome database. Crystal structure datasets of human proteomics were from online databases PDB http://www.rcsb.org/pdb/home/home.do and targetDB (http://targetdb.pdb.org/, downloaded on Mar 25, 2009).

### Functional Annotations Data

Function categories in the PIRSF (http://pir.georgetown.edu/pirsf/, downloaded on Mar 31, 2009), GO (http://www.geneontology.org/, downloaded on Mar 31, 2009), and KEGG (http://www.genome.jp/kegg/, downloaded on Mar 31, 2009) databases were used as source function annotations, whose well-defined categories are widely used for important functional identification analysis. In this study, each candidate gene was annotated onto its corresponding functional families or categories using these three databases.

### Genome-wide Bayesian Association Analysis

We assume here that data *D*, are counts of cases and controls for each of the three genotypes at a SNP locus (Table 1). Bayesian approaches compare the probability of *D *if there is an association (alternate hypothesis *H_1_*) to its probability given no association (null hypothesis *H_0_*). Although most case-control studies are retrospective, we adopted a prospective viewpoint in which a case-control status was the outcome variable and the genotype was regarded as known. Under *H_0_*, the probability of the observed dataset D does not depend on genotype, and can be written in terms of the probability θ that an individual included in the study is a case,

**Table 1:** Frequency of cases and controls for each of three genotypes at a SNP locus.

Genotype:	AA	AB	BB	Total
Case	$n_{0}^{A}$	$n_{1}^{A}$	$n_{2}^{A}$	$n_{0}^{A}+n_{1}^{A}+n_{2}^{A}$
Control	$n_{0}^{U}$	$n_{1}^{U}$	$n_{2}^{U}$	$n_{0}^{U}+n_{1}^{U}+n_{2}^{U}$
Total:	$n_{0}^{A}+n_{0}^{U}$	$n_{1}^{A}+n_{1}^{U}$	$n_{2}^{A}+n_{2}^{U}$	$n_{0}^{A}+n_{1}^{A}+n_{2}^{A}+n_{0}^{U}+n_{1}^{U}+n_{2}^{U}$

Where $n_{0}^{A},n_{1}^{A},n_{2}^{A},n_{0}^{U},n_{1}^{U},n_{2}^{U}$ represents the frequency for each of the specific genotype AA, AB or BB, respectively.

(1) $$P(D/\theta )=c\theta^{n^{A}}{\left(1-\theta \right)}^{n^{U}}$$

where we introduce *n^A ^*and *n^U ^*for the numbers of cases (affected) and controls (unaffected), and *^C ^*is a combinatorial constant that cancels out below and so can be ignored. Here ^θ ^is a "nuisance" parameter, whose value is not important, so under the Bayesian approach we eliminated it by integration with a prior probability distribution. For the purposes of this illustration, the uniform prior is a convenient choice, so

(2) $$P(D)=\int_{0}^{1}P(D/\theta )d\theta =cB(n^{A}+1,n^{U}+1)=B(n^{A}+1,n^{U}+1)$$

where B denotes the Beta function, defined by

$$B(n^{A}+1,n^{U}+1)=\frac{n^{A}!n^{U}!}{(n^{A}+n^{U}+1)!}$$

where *n^A^*! = *n^A ^* × (*n^A^*-1) × (*n^A^*-2) × K × 1.

To compute a probability for D under *H_1_*, we assumed that individuals with genotype *j *had a probability of *θ*_*j *_to be a case. Then, analogous to (1),

(3) $$P(D|\theta_{0},\theta_{1},\theta_{2})=c\theta_{0}^{n_{0}^{A}}{\left(1-\theta_{0}\right)}^{n_{0}^{U}}\times \theta_{1}^{n_{1}^{A}}{\left(1-\theta_{1}\right)}^{n_{1}^{U}}\times \theta_{2}^{n_{2}^{A}}{\left(1-\theta_{2}\right)}^{n_{2}^{U}}$$

where $n_{j}^{A}$ and $n_{j}^{U}$ denote the numbers of cases and controls with genotype *j *= 0, 1, 2. We took the easiest approach first, assuming that each *θ_j _*had an independent, uniform prior, and integrating to obtain [32]

(4) $$P(D)=cB(n_{0}^{A}+1,n_{0}^{U}+1)\times B(n_{1}^{A}+1,n_{1}^{U}+1)\times B(n_{2}^{A}+1,n_{2}^{U}+1)$$

The next step was to compute the Bayes Factor (BF), which is the ratio of (4) to (2). The corresponding formula is:

(5) $$BF=\frac{B(n_{0}^{A}+1,n_{0}^{U}+1)B(n_{1}^{A}+1,n_{1}^{U}+1)B(n_{2}^{A}+1,n_{2}^{U}+1)}{B(n_{0}^{A}+n_{1}^{A}+n_{2}^{A}+1,n_{0}^{U}+n_{1}^{U}+n_{2}^{U}+1)}$$

Values of BF larger than one support *H_1_*, while BF < 1 indicated support for the null H_0_.

To reduce the computational complexity, we used the log value of BF, *BFLn*, as our final function to screen the significant SNP set associated with the disease for each SNP *V_i _*(i = *1,…, N*, where *N *is the total number of SNPs in the GWAS.

(6) $$\begin{array}{l} BFLn(V_{i})=\ln B(n_{0}^{A}+1,n_{0}^{U}+1)+\ln B(n_{1}^{A}+1,n_{1}^{U}+1)+\ln B(n_{2}^{A}+1,n_{2}^{U}+1)\\ \;\;\;\;\;-\ln B(n_{0}^{A}+n_{1}^{A}+n_{2}^{A}+1,n_{0}^{U}+n_{1}^{U}+n_{2}^{U}+1) \end{array}$$

Values of *BFLn*(*V_i_*) larger than zero support *H_1_*, while *BFLn*(*V_i_*) < 0 indicated support for the null *H_0_*.

We then associated gene *g_t _*(t = *1… T*), where T was the number of all genes in the human genome, with SNP *V_i_*, if this SNP was located within g_t _or if g_t _was the closest to *V_i_*. SNPs that were 500 kb from any gene were considered because most enhancers and repressors are <500 kb away from genes, and most linkage disequilibrium blocks are <500 kb away [9]. We carried out first dimensional screening, namely genetic screening, by collecting a test set from genes associated with at least one significant SNP (*BFLn > 0*) and located within one or more disease loci for further filtering.

### SVM Classification based on Sequence and Structure Similarity Features

ID Converter[33] was used to map all genes to their corresponding proteins across the entire human genome. In this section, the positive set consisted of rheumatoid arthritis disease genes from NCBI and the OMIM online database (Additional file 1). The negative set contained the remaining genes that did not fall within any disease loci after excluding genes in the positive set and the test set.

To simplify our analysis, a 28-dimension vector of physicochemical features (Table 2), a combinational pseudo-sequence, was used to represent each protein in positive, negative, and testing sets, according to the online RCSB PDB and targetDB databases. We used 8-dimension secondary features (21-28) and the entire 28-dimension physicochemical features to train two classifiersfor the second screen.

**Table 2:** Protein sequence and structure-based features from PDB and targetDB databases.

Dimension	Feature	Properties
1-20	C	Composition of the 20 amino acid residues
21	a	Cell length a in Angstroms
22	b	Cell length b in Angstroms
23	c	Cell length c in Angstroms
24	alpha	Cell angle alpha in degrees
25	beta	Cell angle beta in degrees
26	gamma	Cell angle gamma in degrees
27	helical	Percent of helical in protein sequence
28	beta sheet	Percent of beta sheet in protein sequence

Considering the diversity of the putative non-disease-candidate proteins, the non-disease-candidate space might not have been sampled completely. Therefore, we constructed 1000 additional training sets (positive:negative = 1:1), in which each negative set was selected randomly from the original negative set. During each randomization, the 8- and 28-dimension features were used to construct the corresponding classifier. The performance of our model was evaluated with an n-fold cross-validation test. In the cross-validation test, the entire positive and negative data sets were shuffled and split into n folds. Each fold was used in turn for testing and the remaining part (n-1 folds) used for training. The sensitivity (*Q_p_*), specificity (*Q_n_*) and overall accuracy (*Q_a_*) were used to measure the accuracy of positive prediction, negative prediction, and the overall accuracy of the model [34], respectively.

$$\begin{array}{l} Q_{p}=\text{TP/(TP + FN)}\\ Q_{n}=\text{TN/(TN + FP)}\\ Q_{a}=\text{(TP + TN)/(TP + TN + FP + FN)} \end{array}$$

Variables are true positives (TP), true negatives (TN), false positives (FP), and false negatives (FN). In general, the overall accuracy *Q_a _*was used to measure the predictive power of a model.

We constructed 10,000 additional training sets (positive:negative = 335:335), in which each negative set was selected randomly from the original negative set. Here, the test set was prepared from genetic screens. We used the training sets, the test set, and the optima classifier to retrain the classifier, aiming to classify the genes in the test set, and then predict candidate genes for the disease. We performed randomization 10,000 times, and kept genes that were judged to be disease genes at each process of randomization.

The SVM used here was Libsvm http://www.csie.ntu.edu.tw/~cjlin/libsvm[35]. The commonly used kernel function, radial basis function (RBF) was introduced into our analysis. According to machine learning theory [36], an optimal hyperplane was drawn by the SVM model to separate positive samples from negative ones. The distance to the hyperplane is related to the confidence of a prediction. Therefore, the distance from each sample to the hyperplane was employed to predict the disease candidate likeness for genes or proteins.

### Functional Annotation Screening and Candidate Gene Prediction

The PRISF, GO, and KEGG pathway databases are widely used for functional studies and gene annotations [37,38]. We hypothesized that disease genes would gather in specific protein families, participate in the same biological functions, or interact within specific biological pathways. According to these three databases, specific biological functions were annotated for known disease genes, and for genes from SVM screening. In this section, we define three functions (*f_PIRSF_, f_GO _*and *f_KEGG_*) to evaluate whether each candidate gene in the set from SVM screening was strongly associated with the disease. The corresponding function formulas were:

$$\begin{array}{c} f_{PIRSF}(g_{i})=\{\begin{array}{ll} 1, & \begin{array}{l} {\text{if g}}_{i}\text{ is annotated onto at least one}\\ \;\text{ protein family that disease genes enriched;} \end{array}\\ 0, & \text{otherwise;} \end{array}\\ f_{KEGG}(g_{i})=\{\begin{array}{ll} 1, & \begin{array}{l} {\text{if g}}_{i}\text{ is annotated onto at least one}\\ \;\;\text{KEGG pathway that disease genes enriched;} \end{array}\\ 0, & \text{otherwise;} \end{array}\\ f_{GO}(g_{i})=\{\begin{array}{ll} 1, & \begin{array}{l} {\text{if g}}_{i}\text{ is annotated onto at least one}\\ \;\;\text{GO term that disease genes enriched;} \end{array}\\ 0, & \text{otherwise;} \end{array}\\ f(g_{i})=f_{PIRSF}(g_{i})\vee f_{GO}(g_{i})\vee f_{KEGG}(g_{i})=\{\begin{array}{ll} 1, & if\;g_{i}\text{ is defined as a candidate;}\\ 0, & \text{otherwise}\text{.} \end{array} \end{array}$$

where *g_i _*is any gene in the resulting set from the first and second screens.

Here, a sample description was listed below for functional annotation screening (Table 3).

**Table 3:** Sample description for functional annotation screening.

_Genes*_	***f***_***PIRSF***_	***f***_***GO***_	***f***_***KEGG***_	***f = f***_***PIRSF ***_***V***_***GO ***_***V***_***KEGG***_
*g_1_*	0	0	0	0
*g_2_*	0	0	1	1
*g_3_*	0	1	1	1
...	...	...	...	...
*g_n_*	1	1	1	1

* contains the genes from SVM screening; columns *f_PIRSF_*, *f_GO_*, *f_KEGG_*, are functional annotation information from PRISF, GO and KEGG databases; '*V*'is an "or" command.

Finally, we used function *f*(*g_i_*) for functional annotation screening by retaining genes (*f*(*g_i_*) = 1) that shared at least one similar functional annotation.

### Comparison with Traditional GWAS

Traditional GWAS analysis [39] uses the Fisher exact test and multiple testing adjustment. Functional enrichments in GO biological processes and KEGG pathways were carried out for known disease genes, GWAS genes, and our predicted genes. Functional consistency with known disease genes was examined to evaluate GWAS genes and our predicted genes. To further evaluate the performance of our screening method, we used the NCBI PubMed module to retrieve associations of GWAS genes or our genes for rheumatoid arthritis using the term "GENE symbols+rheumatoid arthritis" (*e.g*. CD4+rheumatoid arthritis) to determine the underlying mechanisms of the genes from our model.

## Results and Discussions

A genome-wide Bayesian association analysis was carried out to identify variants within genes that were modestly associated with rheumatoid arthritis. The dataset, produced by the Genetic Analysis Workshop (GAW), was 2062 samples genotyped with an Affymetrix Gene Chip Human Mapping 500 K Array Set. Quality control for this dataset included assessment of marker genotype frequency, allelic frequency, and departure from Hardy-Weinberg equilibrium. A total of 433,766 SNPs survived the quality control protocol and were tested for association with the trait in 868 cases and 1194 controls. Significant SNPs (*BFLn > 0*) were mapped onto their corresponding genes, and these genes were considered for further analysis. This process resulted in 4402 candidate risk genes, which were labeled as members of the test set for the SVM screening step.

For the SVM screening, we extracted the sequence and structure information from the PDB and targetDB databases and calculated their feature values for 335 known disease genes, 28,874 non-disease genes, and 4402 other genes in the test set. We used 8-dimension secondary features (21-28) and the entire 28-dimension physicochemical features to train two classifiers for the second screening (see Materials and Methods). To address the concern that, considering the diversity of the putative non-disease-candidate proteins, the non-disease-candidate space might not have been sampled completely, we constructed 1000 additional training sets (positive:negative = 1:1), with each negative set selected randomly from the original negative set. For each randomization, the 8- and 28-dimension features were used to construct the corresponding classifiers. The performance of our model was evaluated with a 5-fold cross-validation test in which the entire positive and negative data sets were shuffled and split into five folds. Each fold was used for testing, and the remaining part (5-1 folds) was used for training. The 1000 randomization results from the two classifiers were analyzed, and the relevant accuracy of 28-dimension physicochemical features varied between 0.695 and 0.891 (Table 4).

**Table 4:** Performance information of two classifiers based on 8-dimension secondary physicochemical features and 28-dimension physicochemical features.

features	prediction	average ± std
8-dimension secondary features(21-28)	0.631-0.831	0.703 ± 0.038
28-dimension physicochemical features	0.695-0.891	0.762 ± 0.036

Based on predictions from these two classifiers, we chose the second classifier for candidate gene prediction. We reconstructed 10,000 additional training sets (positive:negative = 335:335), in which each negative set was selected randomly from the original negative set. The test set was prepared from genetic screens. After 10,000 randomizations, the intersection of each prediction was defined as the final prediction, resulting in 495 candidate genes that used for the third screening step.

We used three functional databases (PRISF, Gene Ontology [GO], and KEGG pathway) to identify responsible risk genes that had similar functional annotations to known disease genes. We hypothesized that disease genes would gather in specific protein families, participate in the same biological functions, and interact in specific biological pathways. According to the three databases, specific biological functions were annotated for 335 known disease genes and 495 genes from the SVM screening. For 495 genes, according to defined function *f(g_i_)*, we collected candidate genes for which each value of their corresponding function *f(g_i_) *equaled 1, demonstrating their strongly functional associations with known disease genes. We identified 146 candidate disease genes as our final candidate predictions for rheumatoid arthritis (Additional file 2).

**Figure 1:**
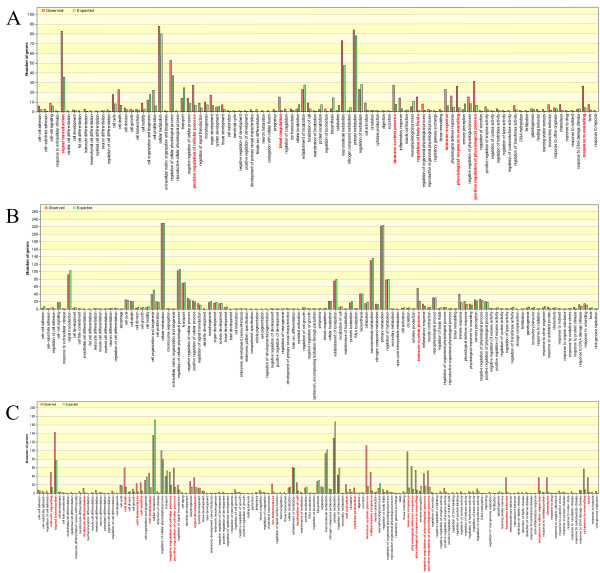
**Results of GO functional enrichment of candidate genes and known disease genes**. (A) GO functional enrichment of candidate genes predicted by our method. (B) GO functional enrichment of candidate genes predicted by the GWAS method. (C) GO functional enrichment of known disease genes.

**Figure 2:**
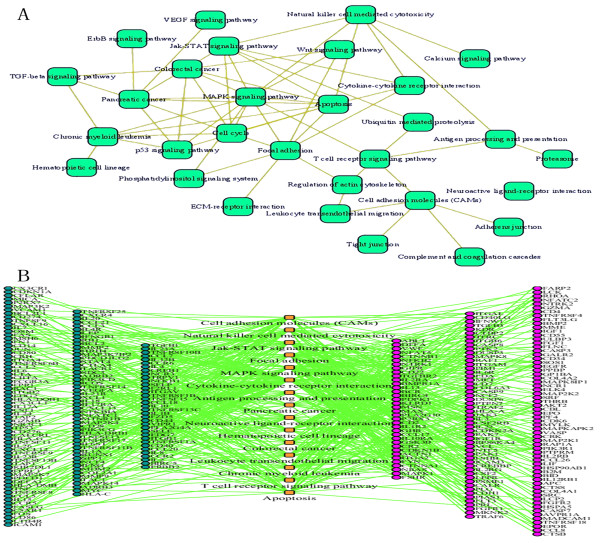
**Pathway-pathway interactions and gene-pathway relationships**. (A) Interactions between pathways of rheumatoid arthritis. Green vertices are responsible pathways and edges are interactions. (B) Relationships between candidate disease genes and corresponding pathways. Orange nodes are responsible pathways, blue nodes are known disease genes, and pink nodes are predicted disease genes.

We used the web software toolkit, Gene Webgetal[40] to investigate the relationship between the 146 candidate genes and the known disease genes, and found several GO functional categories (Figure 1) and pathways in which candidate genes were over-represented, and appeared to interact with other pathways that led to the pathogenesis of the disease. The 146 genes were enriched in signal transduction, positive regulation of cellular process, the immune system and immune response, and the physiological response to wounding, which was coincident with known disease genes (Figure 1). Responsible pathways included cytokine-cytokine receptor interaction pathways, Jak-STAT signaling pathways, cell adhesion molecules, and MAPK signaling pathways (Figure 2; Additional file 3 and 4). Candidate genes and known disease genes not only shared the same pathways, but also linked enriched pathways involved in passing on disease risk (Figure 2).

**Figure 3:**
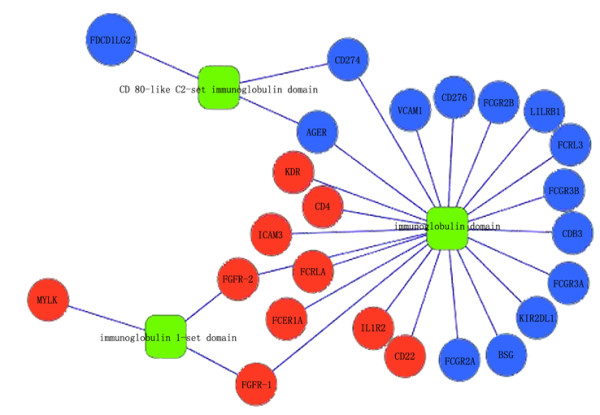
**Known disease genes and predicted genes are enriched in immunoglobulin protein family**. Nodes in blue represents known disease genes, red ones are predicted genes.

The nature of our screening approach meant that many of our predictions overlapped extensively in similar function categories. Therefore, to describe functions representative of association with rheumatoid arthritis, we selected those with the strongest association that also displayed a higher functional enrichment. For example, consistent with all previous studies of rheumatoid arthritis, genes in our gene set included members of the immunoglobulin protein family (Figure 3), the protein kinase domain family, the SH3 domain family, and the ligand-binding domains of nuclear hormone receptor family, and included several genes associated with moderate disease risk, and commonly reported genes such as CD4 [41-44], FGFR1 [45-47], and KDR [48-52]. Genes in the immunoglobulin protein family have a crucial role [53,54] in the pathogenesis of the disease. FGF-2 is transferred to FGFR-1 through binding to HSPG, resulting in RANKL and ICAM-1-mediated maturation of osteoclasts via ERK activation. FGF-2 not only augments the proliferation of RASFs, but is involved in osteoclast maturation, leading to bone destruction in rheumatoid arthritis. Genes in the immunoglobulin protein family also showed strong association with specific biological processes such as receptor binding, protein binding, and molecular transducer activity. Not surprisingly, a KEGG pathway search using these genes yielded the terms "cell adhesion molecules (CAMs)", "cytokine-cytokine receptor interaction" and "Jak-STAT signaling pathway" as the most significantly represented, similar to the enrichment analysis for known disease genes in these pathways. The candidate genes either interacted directly with enzymes associated with known disease genes, or conveyed disease risk indirectly.

We carried out GWAS to find the candidate gene set. The threshold of significant P-value (Bonferroni test) was set at 1.835e-8. GWAS identified 822 candidate genes (Additional file 5). We used Gene Webgetal software to check underlying biological associations for evidence of these candidates in the GO and KEGG databases during rheumatoid arthritis pathogenesis. Compared to traditional GWAS, which is based on multiple testing, we found that few candidate genes overlapped with the results of our multidimensional screening method. Most of the candidate genes in our prediction were verified as modestly associated with rheumatoid arthritis by literature retrieving, but were not identified by a traditional GWAs approach (Additional file 1). We note that a large number of candidate genes from the traditional prediction could not easily be classified into the related functional categories or interacting biological processes associated with this disease. This was not the case for our prediction, demonstrating the effectiveness of our proposed method (Figure 1, Additional file 3, 4 and 6). Candidate genes from GWAS tended to participate in immune systems processes (Figure 1), antigen processing and presentation, glutathione metabolism, cell adhesion molecules (CAMs) and glutathione metabolism and so on (Additional file 6). Even if dysfunction was found in these biological processes, little effect would be expected on other biological processes or pathways, and would not lead to systemic abnormalities or impairment in the function of human essential immune system (Figure 2A). Thus, we propose that the results from strictly statistical methods can find significant candidate genes, but does not consider minor- or medium-risk genes, and this might make uncovering the underlying pathogenesis of rheumatoid arthritis difficult for researchers in the post-genome area. Some candidates from our predicted results lack defined functional descriptions, and require further studies to verify their associations or mechanisms with rheumatoid arthritis, such as NTRK1, IL1R2, and SERPIND1.

Multidimensional approaches can also be applied to candidate gene identification of other diseases, where multiple genes share underlying biological similarities (*e.g*. the same pathway or GO term), or contribute to disease etiology but have common variations that make modest contributions to disease risk. Considering underlying biological similarities together with the proposed method, rather than focusing on a few SNPs or genes with the strongest evidence of disease association can detect likely causal genes. We hope that the proposed method provides additional insights into the pathogenesis of other diseases using hundreds of genetic variance in datasets.

## Conclusions

In this article, we introduce a multi-dimensional screening approach to analyze the 16th Genetic Analysis Workshop (GAW16) data for rheumatoid arthritis, and identify candidate genes for rheumatoid arthritis. Our proposed approach is based on underlying biological similarities-based methods for candidate and known disease genes. Application of our method could identify likely candidate disease genes for rheumatoid arthritis, and could yield biological insights that are otherwise undetectable when focusing only on genes with the strongest evidence by multiple testing.

Traditional GWAS have been developed to identify susceptibility genes assuming a "most significant SNPs/genes" model. This screening process uses a strict selection of statistical thresholds, and aims to identify susceptibility genes based only on the statistical model, without considering multi-dimensional biological similarities in sequence arrangement, crystal structures, and functional categories or biological pathways shared between candidate and known disease genes. Thus, many minor or modestly associated risk genes are likely to be missed after multiple testing adjustments. GWAS and our methods have different objectives. The aim of our method is to avoid arbitrary multiple testing so that more risk biomarkers can be considered. Rather than focusing on individual genes for which evidence is strongest, our multidimensional screening approach typically extracts all risk SNPs/genes (*BFLn *> 0) by their odds ratios for hypothesis *H_1 _*to *H_0 _*, and looks for genes that share underlying biological similarities with known disease genes. We identified multiple genes sharing underlying biological similarities that contributed to disease etiology, but for which common variations made modest contributions to disease risk. A large number of candidate genes from traditional prediction could not be easily classified into related functional categories or interacting biological processes that are associated with the disease.

By considering underlying biological similarities together, rather than focusing on a few SNPs or genes with the strongest evidence of disease association, we can detect likely causal genes using the predicted method. We hope this alternative model complements the most significant SNPs/genes model, and provides additional insights into the pathogenesis of rheumatoid arthritis and other diseases, when using hundreds of genetic variance datasets.

## Competing interests

The authors declare that they have no competing interests.

## Authors' contributions

LCZ and LNC guided the research and analyses described in the paper. LCZ and WL carried out multi-dimensional screening analysis, and LLS participated in performance evaluation of the results. LCZ and LNC participated in coordination of the study. All authors read and approved the final manuscript.

## Pre-publication history

The pre-publication history for this paper can be accessed here:

http://www.biomedcentral.com/1755-8794/3/38/prepub

## Supplementary Material

Additional file 1 **known disease genes collected from the OMIM database and NCBI database**. Click here for file

Additional file 2 **candidate genes with their corresponding risk SNPs predicted by our proposed method and biological evidences between genes and rheumatoid arthritis**. Click here for file

Additional file 3 **the KEGG functional enrichment of candidate genes predicted by our proposed method**. Click here for file

Additional file 4 **the KEGG functional enrichment of known disease genes**. Click here for file

Additional file 5 **candidate genes with their corresponding risk SNPs out of GWAS and their corresponding risk SNPs and biological evidences between genes and rheumatoid arthritis**. Click here for file

Additional file 6 **the KEGG functional enrichment of candidate genes out of GWAS**. Click here for file
